# Benefit of rituximab maintenance is associated with Follicular Lymphoma International Prognostic Index in patients with follicular lymphoma

**DOI:** 10.1097/BS9.0000000000000144

**Published:** 2022-12-09

**Authors:** Ru Li, Tingyu Wang, Rui Lyv, Yi Wang, Ying Yu, Yuting Yan, Qi Sun, Wenjie Xiong, Wei Liu, Weiwei Sui, Wenyang Huang, Huijun Wang, Chengwen Li, Jun Wang, Dehui Zou, Gang An, Jianxiang Wang, Lugui Qiu, Shuhua Yi

**Affiliations:** a State Key Laboratory of Experimental Hematology, National Clinical Research Center for Blood Diseases, Haihe Laboratory of Cell Ecosystem, Institute of Hematology & Blood Diseases Hospital, Chinese Academy of Medical Sciences & Peking Union Medical College, Tianjin 300020, China; b Tianjin Institutes of Health Science, Tianjin 301600, China.

**Keywords:** Follicular lymphoma, Follicular lymphoma international prognostic index, Induction therapy, Maintenance, Progression-free survival, Rituximab

## Abstract

Rituximab maintenance (RM) prolongs the progression-free survival (PFS) of responding patients with follicular lymphoma (FL), but the maintenance efficacy in different Follicular Lymphoma International Prognostic Index (FLIPI) risk group is still confusing. We performed a retrospective analysis of the effect of RM treatments in patients with FL responding to induction therapy based on their FLIPI risk assessment carried out prior to treatment. We identified 93 patients between 2013 and 2019 who received RM every 3 months for ≥4 doses (RM group), and 60 patients who did not accept RM or received rituximab less than 4 doses (control group). After a median follow-up of 39 months, neither median overall survival (OS) nor PFS was reached for the entire population. The PFS was significantly prolonged in the RM group compared to the control group (median PFS NA vs 83.1 months, *P* = .00027). When the population was divided into the 3 FLIPI risk groups, the PFS differed significantly (4-year PFS rates, 97.5% vs 88.8% vs 72.3%, *P* = .01) according to group. There was no significant difference in PFS for FLIPI low-risk patients with RM compared to the control group (4-year PFS rates, 100% vs 93.8%, *P* = .23). However, the PFS of the RM group was significantly prolonged for FLIPI intermediate-risk (4-year PFS rates, 100% vs 70.3%, *P* = .00077) and high-risk patients (4-year PFS rates, 86.7% vs 57.1%, *P* = .023). These data suggest that standard RM significantly prolongs the PFS of patients assigned to intermediate- and high-risk FLIPI groups but not to low-risk FLIPI group, and pending larger-scale studies to validate.

## 1. INTRODUCTION

Follicular lymphoma (FL) is the one of common lymphomas in the western countries,^1,2^ and accounts for 2.9% to 3.9% of all lymphoid neoplasms in China.^3,4^ FL is an incurable, indolent disease characterized by repeated relapses.^5^ With the advent of rituximab immunotherapy in 1997, significant improvements have been made in lymphoma and other cancer therapy over the past 2 decades, which has changed the natural course of the disease and its treatment regimens. Consequently, rituximab-based immunochemotherapy is now the standard choice for first-line therapy of FL,^6,7^ and patients can typically expect response rate of nearly 90%, and 5-year overall survival (OS) rates approaching 90%.^8^

Several studies have explored the outcomes of rituximab maintenance (RM) treatment for patients responding to induction. These studies have shown that RM prolongs the progression-free survival (PFS) of patients with untreated FL and relapsed FL^9–11^; however, patients with FL have heterogeneous outcomes regardless of RM treatment. This might be due to differences in individual FL clinical characteristics. There were a few prognostic systems for patients with FL including the Follicular Lymphoma International Prognostic Index (FLIPI), FLIPI-2, m7-FLIPI, and PRIMA-PI.^12–14^ FLIPI is the most commonly used system to stratify patients according to their clinical features and to predict survival.^15,16^ How RM affects patients in different FLIPI risk groups remains unclear. Here, we aimed to evaluate the effectiveness of RM in patients belonging to the 3 FLIPI risk groups to see whether all patients in different FLIPI risks benefit from RM treatments. To do so, we performed a retrospective analysis of the effect of RM treatments in patients with FL responding to induction therapy based on the FLIPI risk assessment carried out prior to treatment.

## 2. METHODS

### 2.1. Patients

**Figure 1.**
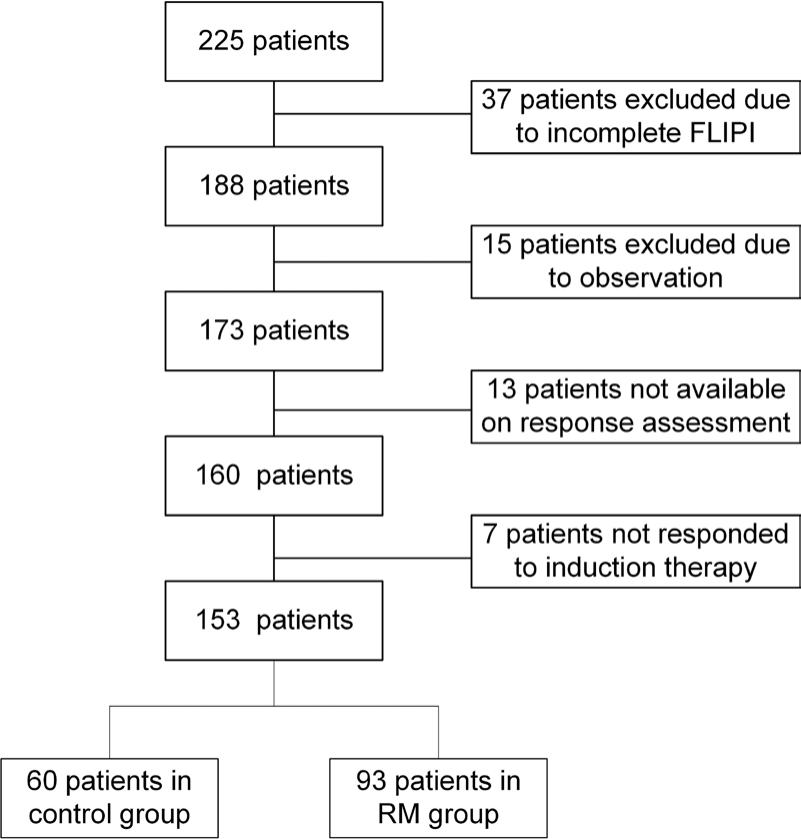
Full details of patients enrolled in this analysis. FLIPI = Follicular Lymphoma International Prognostic Index, RM = rituximab maintenance.

This single-center retrospective study was conducted between January 2013 and December 2019 at the Institute of Hematology & Blood Diseases Hospital, Tianjin, China. A total of 173 adult patients with newly diagnosed FL completed induction therapy with rituximab-combination were identified (**Fig. 1**). All cases involved in this study were approved by the institutional ethics committees (IIT2020023-EC-1), and patient informed consent was obtained in accordance with the Declaration of Helsinki. Eligibility criteria included: (i) patients newly diagnosed with FL according to the criteria of the World Health Organization classification of tumors of hematopoietic and lymphoid tissues in 2013,^17^ and (ii) histological grade 1–3A. Exclusion criteria were as follows: (i) histological grade 3B or histological transformation into diffuse large B cell lymphoma (DLBCL), (ii) incomplete FLIPI score.

Baseline characteristics, including age, sex, laboratory examinations, imaging examinations, and Ann Arbor stage, were collected from individual patient records. Five factors were included for FLIPI: age (>60 vs <60 years), Ann Arbor stage (III–IV vs I–II), hemoglobin level (<120 vs >120 g/L), number of nodal areas (>4 vs ≤4), and serum LDH level (above normal vs normal or below) Three risk groups were defined: low risk (0–1 adverse factor), intermediate risk (2 factors), and high risk (>3 adverse factors).

### 2.2. Treatment regimens and response monitoring

The induction regimen was based on the physicians’ choice. The most common regimen was rituximab, cyclophosphamide, vincristine, doxorubicin, and prednisone (R-CHOP). Other regimens include rituximab, cyclophosphamide, vincristine; doxorubicin, and dexamethasone (R-Hyper-CVAD), the rituximab, etoposide, doxorubicin, cyclophosphamide, vincristine, and prednisone (DA-EPOCH-R), the rituximab, fludarabine, cyclophosphamide, and mitoxantrone (R-FCM), and the bendamustine plus rituximab regimen (BR). Some patients combined rituximab and lenalidomide with chemotherapy such as R2-CHOP and R2-DA-EPOCH. Patients responding to induction therapy received rituximab treatments every 3 months as maintenance therapy, or observation based on the advice of physicians and the choice of patients.

The response was evaluated within 1 to 3 months after the end of the induction therapy through (18)F-fluorodeoxyglucose positron emission tomography (FDG-PET) or computed tomography (CT) plus bone marrow biopsy if the bone marrow was involved at diagnosis according to the 2014 Lugano Classification.^18^ The response after induction therapy included complete response (CR), partial response (PR), stable disease (SD), progressive disease (PD). PFS was defined as the duration from the time of initiation of induction treatment to progression. OS was defined as the time of initiation of induction treatment to death.

### 2.3. Statistical analysis

Categorical variables were compared using Fisher exact tests. Continuous variables were compared by the Wilcoxon rank-sum test. PFS and OS were estimated using the Kaplan-Meier method and comparisons between groups were made by the log-rank test. Statistical analyses were conducted using R version 4.1.2 (https://www.r-project.org/).

## 3. RESULTS

### 3.1. Baseline characteristics

The clinical characteristics of the 173 patients are shown in Table 1. The median age at diagnosis was 47 years old (range 19–70) and 79 patients (45.7%) were male. The bone marrow was involved in most cases (116 patients, 67.1%) and most of the patients (155 patients, 90.1%) had advanced-stage disease (90.1%). The patients were assigned to 1 of 3 risk groups prior to receiving treatment according to FLIPI, 46 (26.6%) patients were in the low-risk group, 82 patients (47.4%) in the intermediate-risk group, and 45 (26.0%) patients in the high-risk group.

**Table 1:** Baseline characteristics.

Characteristic	Level	Control group N (%)	RM group N (%)	*P* value*
Number		60	93	
Age (median [IQR])		49 [40, 55]	43 [38, 52]	.151
Age	≤60	53 (88.3)	86 (92.5)	.562
	>60	7 (11.7)	7 (7.5)	
Sex	Male	31 (51.7)	38 (40.9)	.252
	Female	29 (48.3)	55 (59.1)	
B symptoms	Negative	42 (70.0)	73 (78.5)	.319
	Positive	18 (30.0)	20 (21.5)	
β2MG	Normal	27 (56.2)	46 (59.0)	.908
	Elevated	21 (43.8)	32 (41.0)	
LDH	Normal	54 (90.0)	79 (84.9)	.509
	Elevated	6 (10.0)	14 (15.1)	
Bulky disease	Negative	33 (86.8)	52 (80.0)	.54
	Positive	5 (13.2)	13 (20.0)	
BM involvement	Negative	19 (31.7)	34 (36.6)	.655
	Positive	41 (68.3)	59 (63.4)	
Ann Arbor stage	Early	9 (15.3)	6 (6.5)	.135
	Advanced	50 (84.7)	87 (93.5)	
FLIPI score	Low	19 (31.7)	23 (24.7)	.497
	Intermediate	28 (46.7)	43 (46.2)	
	High	13 (21.7)	27 (29.0)	
EOI response	CRR	47 (83.9)	72 (80.0)	.707

β2-MG = β2-microglobulin, B symptoms = fever, weight loss, night sweats, BM = bone marrow, CRR = complete response rate, EOI = end of induction, FLIPI = Follicular Lymphoma International Prognostic Index, LDH = lactate dehydrogenase, RM = rituximab maintenance, IQR = interquartile range.

* Fisher exact test of frequency between control group and RM group.

### 3.2. Clinical outcomes

#### 3.2.1. Response

A response assessment was available for 160 patients after induction therapy: 153 patients (95.6%) responded to the induction therapy, with a complete response rate (CRR) seen in 119 patients (76.8%). The overall response rate (ORR) for patients considered low, intermediate, and high risk according to the FLIPI was 97.7%, 94.7%, and 95.2%, respectively, and did not differ according to group (*P* = .789). Similarly, the CRR according to the low, intermediate, and high risk according to the FLIPI did not differ by groups, at 88.1%, 72.2%, and 73.2%, respectively (*P* = .125).

#### 3.2.2. Survival

##### 3.2.2.1. Survival of the entire cohort of patients with FL

**Figure 2.**
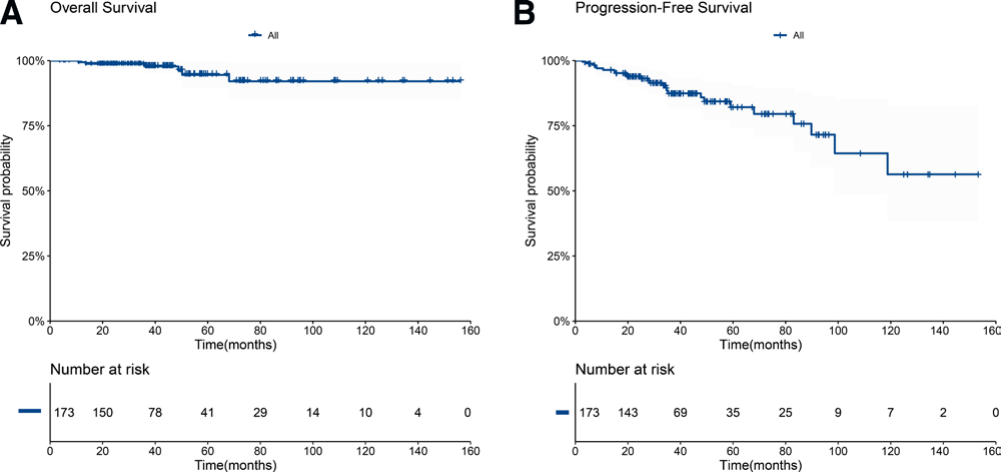
Kaplan-Meier curves showing overall survival (A) and progression-free survival (B) for all patients in the cohort.

**Figure 3.**
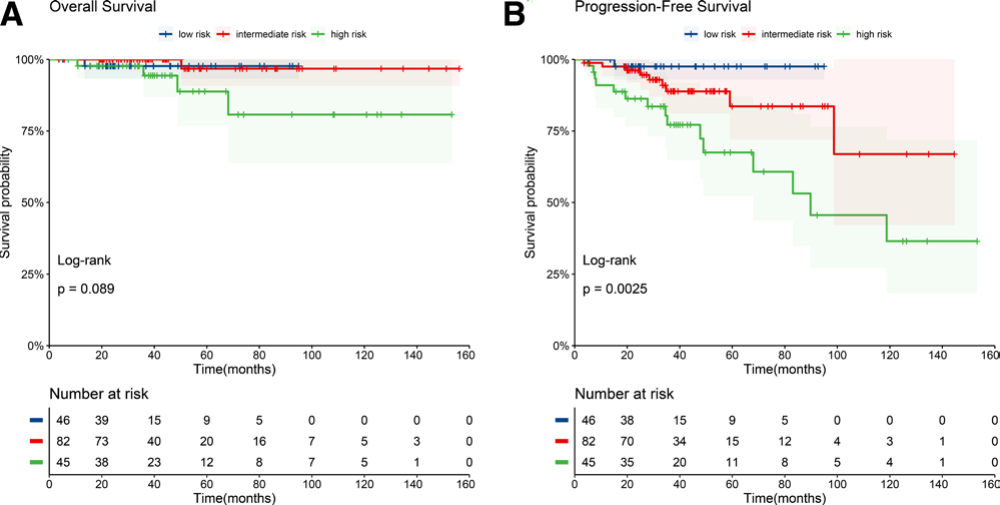
Kaplan-Meier curves showing overall survival (A) and progression-free survival for patients stratified by FLIPI risk group. FLIPI = Follicular Lymphoma International Prognostic Index.

We achieved a median follow-up of 39 months (range, 4–156 months) across our eligible patient cohort. The median OS and PFS were not reached in the entire population. At 4 years, the OS for the entire cohort was 97.8% (95% confidence interval [CI], 95.2%–100%), and the PFS was 85.9% (95% CI, 79.7%–92.5%; **Fig. 2**). The median OS for patients in the low-, intermediate-, and high-risk FLIPI groups was not reached, and 4-year OS rates were 97.7% (95% CI, 93.3%–100%), 100%, and 94.4% (95% CI, 86.9%–100%), respectively. There was no difference in the 4-year OS between the groups (*P* = .2 **Fig. 3A**). The median PFS was not reached for patients in FLIPI low- and intermediate-risk groups, but was 89.8 months (95% CI, 68–NA) for patients in the FLIPI high-risk group. The 4-year PFS rates were 97.6% (95% CI, 93.0%–100%) for patients in the low-risk group, 88.8% (95% CI, 81.2%–97.2%) for patients in the intermediate-risk group, and 72.3% (95% CI, 58.3%–89.7%) for patients in the high-risk group. We saw a statistically significant difference in 4-year PFS between FLIPI risk groups (*P* = .01, **Fig. 3B**).

##### 3.2.2.2. Survival according to RM status

Of the 153 patients who responded to the induction therapy, 60 patients did not receive RM treatment or received fewer than 4 doses (control group). The other 93 patients received RM more than 4 times (RM group). The median number of rituximab treatments in the RM group was 7 (range 4–10). Most patients discontinued RM treatment due to financial reasons. The 2 groups were well matched in terms of baseline characteristics such as age, sex, Ann Arbor stage, lactate dehydrogenase (LDH) levels, and FLIPI score (Table 1).

**Figure 4.**
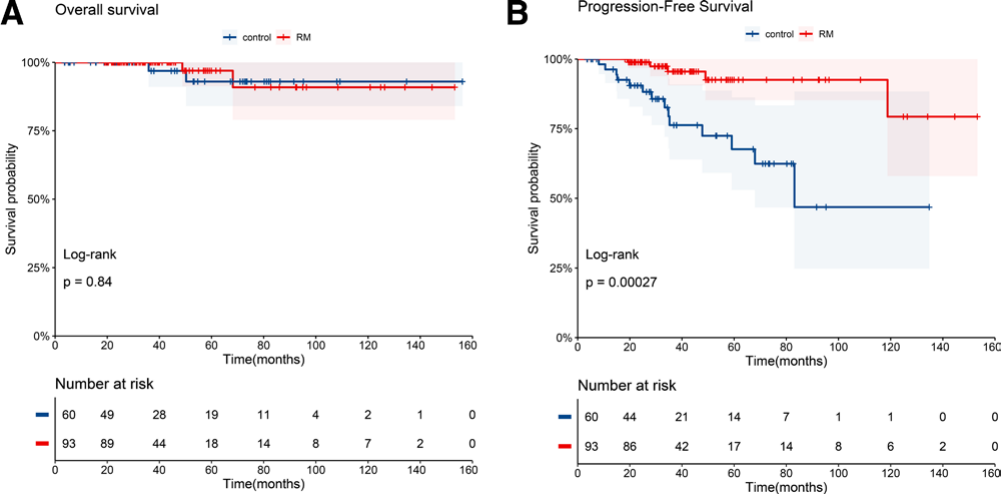
Kaplan-Meier curves showing progression-free survival (A) and overall survival (B) of patients by duration of RM treatment. RM = rituximab maintenance.

PFS was significantly prolonged in the 93 patients in the RM group compared with the control group (*P* = .00027). Specifically, the median PFS was 83.1 months (95% CI, 68.0–NA) for the control group, and was not reached for the RM group (95% CI, NA–NA) (**Fig. 4A**). The 4-year PFS rate was 72.4% (95% CI, 59.2%–88.7%) for patients in the control group and 95.5% (95% CI, 90.6%–100%) for patients in the RM group (*P* = .0007). Despite this prolongation in the PFS, we saw no difference in OS between the 2 groups (**Fig. 4B**). The follow-up of 120 patients were longer than 24 months. Among them, there were 44 patients in the control group and 4 (9.1%) patients experienced progression within 24 months (POD24). And there were 76 patients in the RM group, 1 (1.3%) patient underwent POD24. There were apparently less patients experienced progression in the RM group (*P* = .025).

**Figure 5.**
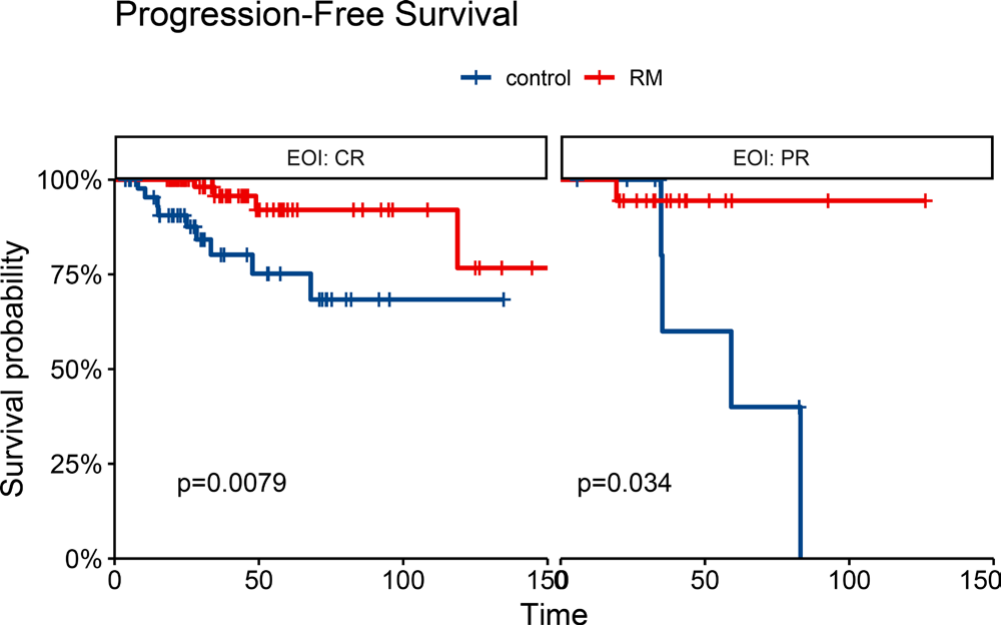
Progression-free survival of patients achieving CR (A) and PR (B) by RM. Control: no rituximab maintenance group. CR = complete response, EOI = end of induction, PR = partial response, RM = rituximab maintenance group.

For patients responding to the induction therapy, there were 119 patients achieved CR, and 34 patients achieved PR. Among 119 patients who achieved CR, the 4-year PFS rates were 75.2% (95% CI, 60.8%–93.9%) in control group and 95.7% (95% CI, 90.0%–100%) in the RM group (**Fig. 5A**). Among 34 patients who achieved PR, the 4-year PFS rates were 60.0% (95% CI, 29.3%–100%) in the control group and 94.4% (95% CI, 84.4%–100%) in the RM group (**Fig. 5B**). Finally, the prolongation in the PFS between those who achieved CR (*P* = .0079) or PR (*P* = .034) was statistically significant.

##### 3.2.2.3. The impact of RM treatment on patients at different FLIPI risks

**Figure 6.**
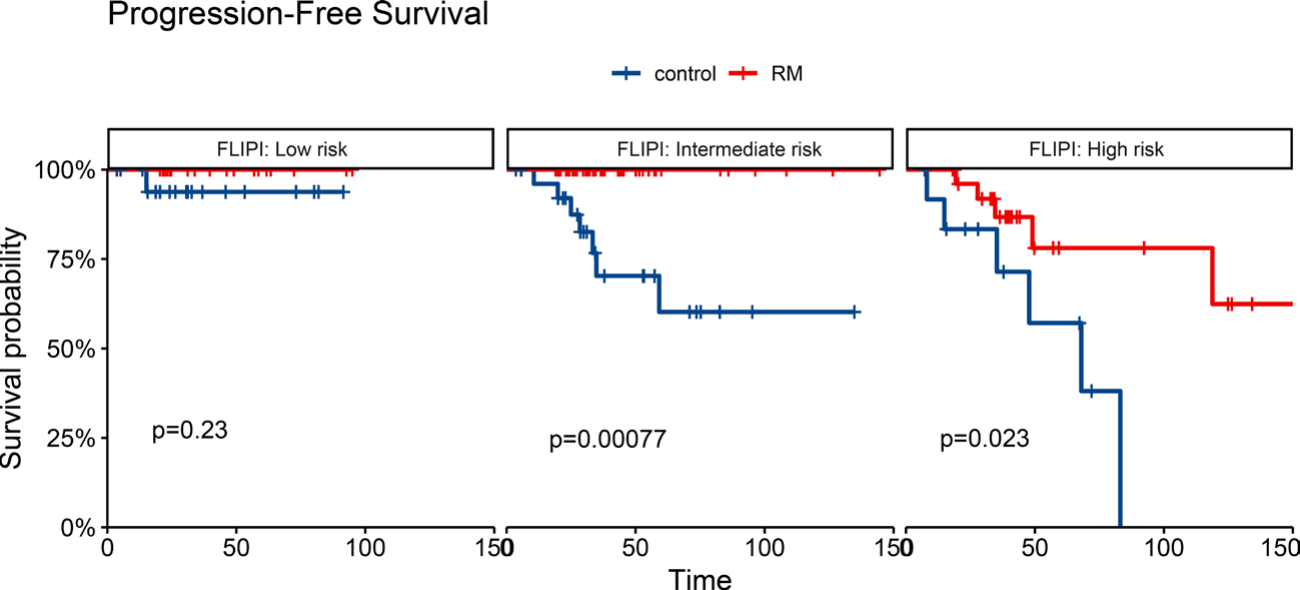
Progression-free survival by RM in the 3 FLIPI risk groups, low risk (A), intermediate risk (B), and high risk (C). FLIPI = Follicular Lymphoma International Prognostic Index, RM = rituximab maintenance.

To determine the efficacy of RM treatment based on the pre-treatment FLIPI risk assessment, the PFS of patients in the RM and control groups after induction were analyzed according to their FLIPI score (**Fig. 6**). For patients categorized as being FLIPI low risk, the 4-year PFS rate was 100% in the RM group (*P* = .2) and 93.8% (95% CI, 82.6%–100%) in the control group. Patients with FLIPI intermediate-risk scores who received RM had a 100% 4-year PFS rate, which was significantly higher than that of the control group, 70.3% (*P* = .002, 95% CI, 52.4%–94.2%). Finally, the FLIPI high-risk patients had similar 4-year PFS rates of 86.7% (95% CI, 73.7%–100%) and 57.1% (95% CI, 31.7%–100%) in the RM and control group, respectively (*P* = .023). The overall PFS for intermediate patients (*P* = .00077) and high-risk patients (*P* = .023) who underwent RM was significantly improved over the control group; however, there was no statistical difference (*P* = .23) for patients in the FLIPI low-risk group.

## 4. DISCUSSION

FL is an indolent disease that tends to respond well to immunochemotherapy. However, patients with FL have heterogenous outcomes. To our knowledge, this is the first evaluation in a Chinese cohort of effects of RM treatment in newly diagnosed patients with FL who responded to induction therapy. This study showed the significance of a RM treatment strategy guided by a pre-treatment FLIPI assessment. The results indicated that patients with intermediate- and high-risk FLIPI scores benefited from RM treatment in terms of PFS while patients with low-risk FLIPI scores did not.

In our center, the ORR was 95.6% and CRR was 76.8% at end of induction therapy for all eligible patients with FL. Validating that our cohort is generally representative of responses in the rituximab era, the response rates in the current study are similar to those identified by Luminari et al in their FOLL12 study (ORR 96%, CRR 82%).^19^ The induction regimens in the FOLL12 study included R-CHOP and BR regimens.

Patients with FL often experience recurrent relapses and unfortunately, the response duration and survival time shortens after each relapse.^20^ Moreover, an early relapse is predictive of a high risk for death.^21^ Patients who experienced progression within 24 months had poor outcomes.^22^ Taking these factors into consideration, treatment regimens must be designed that can prolong the duration of response and postpone or even prevent relapses, especially early relapse. Over the past 2 decades, we have seen that the addition of rituximab to chemotherapy has improved the response and survival rate of patients with FL as rituximab can remove residual FL tumor cells which may lead to relapse.^23,24^ For this reason, rituximab is considered the first-choice maintenance therapy. In line with previous randomized controlled trial PRIMA^9,25^ and a retrospective study with BR as induction regimen,^26^ we found that RM treatment significantly prolonged the PFS for patients who responded to induction therapy, even though the baseline characteristics between patients at our center and PRIMA were different. Specifically, the patients at our center and eligible for our study were younger than those in PRIMA (median age, 47 vs 56 years), but this was consistent with clinical features of 1845 patients in the Chinese multicenter study.^27^ In addition, more patients at our center presented with bone marrow involved (67.1% vs 55.0%) and elevated β2 microglobulin (43.5% vs 32.0%) than those in the PRIMA study. Patients at our center were representative of Chinese patients with FL except for higher BM involvement which may be due to the higher rate of usage of flow cytometer technology in bone marrow aspirate at our hospital. Despite these differences, we saw a significant reduction in the risk of progression for the patients with CR or PR, which was consistent with the results of the FOLL12 study.^19^ Importantly, we found that RM significantly reduced the rate of POD24. But OS was not prolonged due to small sample. It is the first time that RM has been found to reduce the incidence of POD24. Validation is needed in the future.

In most studies, RM was scheduled every 8 weeks. However, the pharmacodynamics study of rituximab showed that CD20+ B cells in the peripheral blood remained depleted for at least 2 to 3 months.^28^ Besides, RM every 2 months is more toxic than every 3 months.^29^ Take these into account, we recommended RM every 3 months. Regarding the optimal duration of RM treatments, our data confirmed that inclusion of RM on the schedule for ≥4 times effectively prolonged the 4-year PFS by up to 95.5%. Importantly, data from a previous study demonstrated that 5-year RM therapy does not improve event-free survival but increases toxicity, thus resulting in infections and lower life quality.^30^ Based on these findings, we consider that the optimal duration of RM treatment is 1 to 2 years to achieve maximal efficacy and minimal toxicity; however, results of our cohort did not contain the adverse events during and after the RM treatments. The toxicity of RM should be taken into consideration in the follow-up in the future studies.

The FLIPI score was developed to risk-stratify patients and to predict survival in the pre-rituximab era.^15^ According to our cohort and previous studies,^16^ the FLIPI score remains a good predictor for PFS in the rituximab era (*P* = .0025). Our subgroup analysis showed no statistical benefit of RM treatment for patients in the FLIPI low-risk group. These findings differ from those of the PRIMA study which showed a PFS benefit of RM in all FLIPI risk groups.^25^ And in FOLL12 study, the patients were assigned into 2 groups according to FLIPI score, those are low-intermediate risk and high risk. Patients in both groups benefited from standard RM treatment in terms of PFS.^19^ However, patients in the FLIPI low-risk group included in this study achieved long PFS irrespective of RM treatment, with approximately 94% of patients in the FLIPI low-risk group still in remission after 4 years. As a common toxic effect of R is lymphocytopenia, which can lead to infection, we would not suggest patients in the low-risk group to undergo RM treatment based on our data. These patients will also benefit from reduced costs and duration of hospitalization by not undergoing, in our opinion, unnecessary RM treatment. Further clinical trials designed to evaluate RM treatments for patients in FLIPI low risk are needed to demonstrate the effects of RM. Patients in our center were much younger and could undertake intensified regimens, like R-EDOCH, and some patients received lenalidomide for leukemic phase, which could prolong the PFS and reduce the progression rate. Here, we saw that the PFS of patients at intermediate and high risks were significantly prolonged with RM treatment. We thus recommend that patients at intermediate and high risk undergo RM treatment. FLIPI-2 and PRIMA-PI were built in the rituximab era. They both worked well in stratifying the patients with PFS. However, β2MG test was not performed routinely at our center before 2014. As a result, FLIPI-2 and PRIMA-PI were not available for part of the patients. In addition, the diameter of nodal disease was missing for near one third of patients which is one of the FLIPI-2 factors. Consequently, FLIPI-2 and PRIMA-PI were not used to determine the effect of RM.

There were limitations. First of all, this study is a retrospective study, the inherent limitations include selection, confounding variables, recall biases, and nonuniform follow-up also affect the results. Thirty-seven patients were excluded from this study because of incomplete FLIPI component which might create a selection bias. As we concerned, the baseline and induction therapies were comparable to the patients enrolled in this study. Second, the induction therapies varied between patients which might influence the results. In real-world practice, the induction therapies were adjusted considering the physical and financial conditions in China.

## 5. CONCLUSION

In summary, our data suggest that to improve PFS and prevent relapse, RM be included on the schedule for a minimum of 1 year for patients with FL whose pre-treatment clinical and biochemical indices indicate that they fall into the FLIPI intermediate- and high-risk groups, and they respond to immunochemotherapy induction. With the duration of remission prolonged, these patients would have more opportunities to receive new target therapies in the future.

## ACKNOWLEDGMENTS

This work was supported by grants from the National Natural Science Foundation of China (81970187, 82170193, 81920108006, and 81900203), Chinese Academy Medical Sciences Innovation Fund for Medical Sciences (2021-I2M-C&T-B-081).

## AUTHOR CONTRIBUTIONS

L.Q. and S.Y. contributed equally to this work.
